# *Sincell*: an R/Bioconductor package for statistical assessment of cell-state hierarchies from single-cell RNA-seq

**DOI:** 10.1093/bioinformatics/btv368

**Published:** 2015-06-22

**Authors:** Miguel Juliá, Amalio Telenti, Antonio Rausell

**Affiliations:** ^1^Vital-IT group, SIB Swiss Institute of Bioinformatics, 1015 Lausanne, Switzerland,; ^2^University of Lausanne, 1015 Lausanne, Switzerland and; ^3^J. Craig Venter Institute, La Jolla, CA 92037

## Abstract

**Summary:** Cell differentiation processes are achieved through a continuum of hierarchical intermediate cell states that might be captured by single-cell RNA seq. Existing computational approaches for the assessment of cell-state hierarchies from single-cell data can be formalized under a general framework composed of (i) a metric to assess cell-to-cell similarities (with or without a dimensionality reduction step) and (ii) a graph-building algorithm (optionally making use of a cell clustering step). The *Sincell* R package implements a methodological toolbox allowing flexible workflows under such a framework. Furthermore, *Sincell* contributes new algorithms to provide cell-state hierarchies with statistical support while accounting for stochastic factors in single-cell RNA seq. Graphical representations and functional association tests are provided to interpret hierarchies. The functionalities of *Sincell* are illustrated in a real case study, which demonstrates its ability to discriminate noisy from stable cell-state hierarchies.

**Availability and implementation:** *Sincell* is an open-source R/Bioconductor package available at http://bioconductor.org/packages/sincell. A detailed manual and a vignette are provided with the package.

**Contact:** antonio.rausell@isb-sib.ch

**Supplementary information**: Supplementary data are available at *Bioinformatics* online.

## 1 Introduction

Unbiased profiling of individual cells through single-cell RNA-seq allows assessing heterogeneity of transcriptional states within a cell population (Wu *et al.*, 2014). In the context of a cell population’s differentiation or activation process, such transcriptional heterogeneity might reflect a continuum of intermediate cell states and lineages resulting from dynamic regulatory programs. Such a continuum might be captured through the assessment of cell-state hierarchies, where each cell is placed in a relative ordering in the transcriptional landscape. Additionally, statistical support should be provided to discriminate reliable hierarchies from stochastic heterogeneity, arising from both technical and biological factors.

**Fig. 1.**
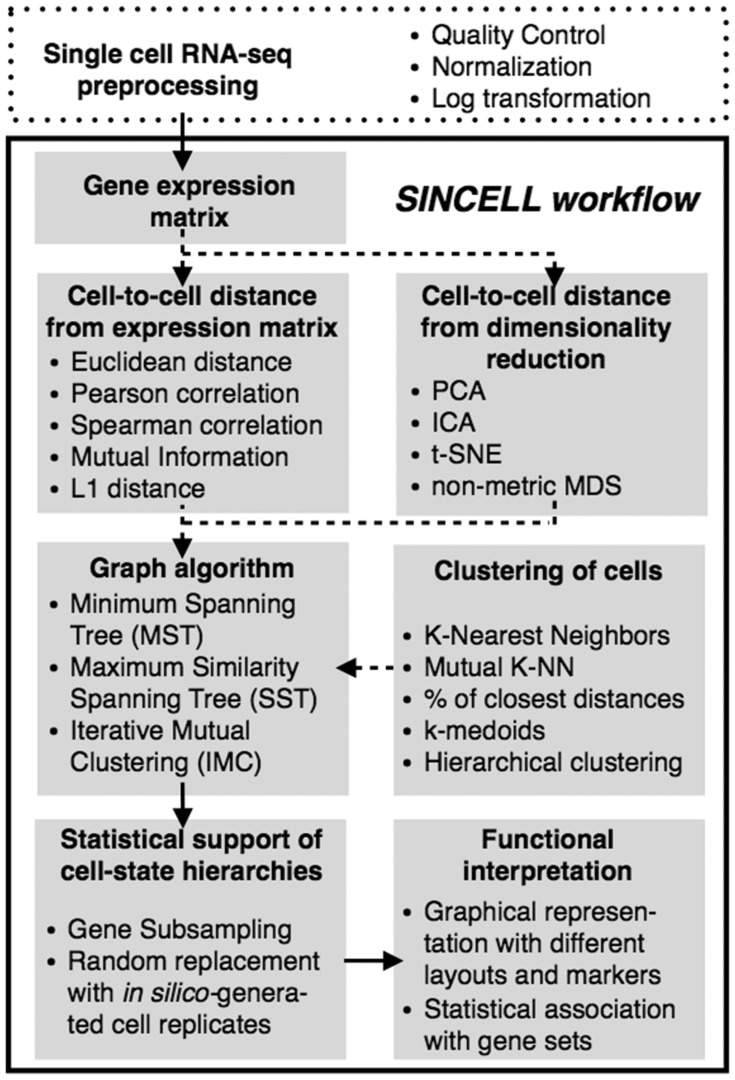
Overall workflow for the statistical assessment of cell-state hierarchies implemented by the *Sincell* R package. Dashed arrows correspond to optional steps in the analysis

A number of algorithms have been used to assess cell-state hierarchies from single-cell data (Amir *et al.*, 2013; Bendall *et al.*, 2014; Buettner *et al.*, 2015; Jaitin *et al.*, 2014; Moignard *et al.*, 2015; Qiu *et al.*, 2011; Trapnell *et al.*, 2014). These approaches can be formalized under a general framework (Supplementary Table S1). Here we present *Sincell*, an R/Bioconductor package where the various building blocks of that general workflow are extended and combined (Fig. 1). Notably, *Sincell* implements algorithms to provide statistical support to the cell-state hierarchies derived from single-cell RNA-seq. The package is complemented with graphical representations and functional association tests to help interpret the results.

## 2 Description

As input, *Sincell* requires an expression matrix with user-defined normalized gene expression levels per single cell (Fig. 1). Variance stabilization is recommended (e.g. through log transformation). First, a cell-to-cell distance matrix is calculated using a metric of choice. *Sincell* provides both linear and non-linear distances: Euclidean, Mutual Information, L1 distance, Pearson and Spearman correlation. Optionally, the distance matrix may be obtained from the leading dimensions of a data reduction technique, performed to keep the most informative parts of the data while excluding noise. Both linear and non-linear algorithms are provided: Principal Component Analysis, Independent Component Analysis, t-Distributed Stochastic Neighbor Embedding and non-metric Multidimensional Scaling.

Second, a cell-state hierarchy is obtained by applying a graph-building algorithm on the cell-to-cell distance matrix. Graph-building algorithms may consider cells both individually and in clusters of highly similar cells. S*incell* provides different clustering methods (K-Mutual Nearest Neighbors, k-medoids, agglomerative clustering, etc.) and graph-building algorithms (MST, SST and IMC; Supplementary Text).

Stochastic technical and biological factors may drive cell-state heterogeneity observed in single-cell RNA seq data. Additionally, hierarchies derived from experiments with a low number of individual cells (e.g. 96 cells when using a Fluidigm C1^™^ Single-Cell Auto Prep System) are more susceptible to noise artifacts than experiments profiling thousands of individual cells (e.g. flow cytometry data). *Sincell* implements two algorithms to discriminate reliable hierarchies from noise-driven ones. The first strategy relies on a gene resampling procedure. The second one is based on random cell substitution with *in silico*-generated cell replicates. These replicates are built by perturbing observed gene expression levels with random noise, following patterns of stochasticity observed in single-cell RNA-seq (Supplementary Text). Either approach generates a population of hierarchies whose similarities to the reference show the distribution of the hierarchy stability against changes in the data.

To help interpret hierarchies in functional terms, *Sincell* provides graphical representations of cell-to-cell similarities in low-dimensional space as well as different graph displays of hierarchies, coloring cells, e.g. by expression levels of a marker of choice. Furthermore, *Sincell* implements an algorithm to determine the statistical significance of the association of the hierarchy with the expression levels of a given gene set (Supplementary Text). Gene set collections (e.g. Gene Ontology terms) can be systematically evaluated.

## 3 Application

The *Sincell* R package includes a detailed vignette illustrating all functionalities using real single-cell RNA-seq data. We use data from (Trapnell *et al.*, 2014) quantifying gene expression levels in differentiating myoblast at 0, 24, 48 and 72 h. Here, we analyze each time-point separately and evaluate the statistical evidence of cell-state heterogeneity within them (Supplementary Fig. 1). Our results show that early differentiation timepoints produce unstable hierarchies suggesting a low level of cell-state heterogeneity. However, late differentiation timepoints produce statistically significant hierarchies that reflect cell-state diversity along the differentiation process (Supplementary Text).

## 4 Discussion

The landscape of computational approaches to assess cell-state hierarchies from single-cell data is far from being fully explored. The diversity of biological studies and rapid evolution of single-cell technologies require a comprehensive toolbox where users may easily tailor workflows and compare alternative methods and assumptions. Furthermore, cell-state hierarchies should be statistically supported before being used as input in subsequent analyses. The *Sincell* R package addresses these needs by providing a general analysis framework, new algorithms for statistical support as well as tools for functional interpretation of cell-state hierarchies.

## Funding

This research was supported by the European Union's Seventh Framework Programme FP7/2007-2013/ under grant agreement no. 305762 and the Swiss National Science Foundation grant no. 149724. Computations were performed at the Vital-IT (http://www.vital-it.ch) Center for high-performance computing of the SIB Swiss Institute of Bioinformatics.

*Conflict of interest*: none declared.

## Supplementary Material

Supplementary Data

## References

[btv368-B1] Amir E.D. (2013) viSNE enables visualization of high dimensional single-cell data and reveals phenotypic heterogeneity of leukemia. Nat. Biotechnol., 31, 545–552. 23685480 10.1038/nbt.2594 PMC4076922

[btv368-B2] Bendall S.C. (2014) Single-cell trajectory detection uncovers progression and regulatory coordination in human B cell development. Cell, 157, 714–725. 24766814 10.1016/j.cell.2014.04.005 PMC4045247

[btv368-B3] Buettner F. (2015) Computational analysis of cell-to-cell heterogeneity in single-cell RNA-sequencing data reveals hidden subpopulations of cells. Nat. Biotechnol., 33, 155–160. 25599176 10.1038/nbt.3102

[btv368-B4] Jaitin D.A. (2014) Massively parallel single-cell RNA-seq for marker-free decomposition of tissues into cell types. Science, 343, 776–779. 24531970 10.1126/science.1247651 PMC4412462

[btv368-B5] Moignard V. (2015) Decoding the regulatory network of early blood development from single-cell gene expression measurements. Nat. Biotechnol., 33, 269–276. 25664528 10.1038/nbt.3154 PMC4374163

[btv368-B6] Qiu P. (2011) Extracting a cellular hierarchy from high-dimensional cytometry data with SPADE. Nat. Biotechnol., 29, 886–891. 21964415 10.1038/nbt.1991 PMC3196363

[btv368-B7] Trapnell C. (2014) The dynamics and regulators of cell fate decisions are revealed by pseudotemporal ordering of single cells. Nat. Biotechnol., 32, 381–386. 24658644 10.1038/nbt.2859 PMC4122333

[btv368-B8] Wu A.R. (2014) Quantitative assessment of single-cell RNA-sequencing methods. Nat. Methods, 11, 41–46. 24141493 10.1038/nmeth.2694 PMC4022966

