# Supplementary material for: Sincell: an R/Bioconductor package for statistical assessment of cell-state hierarchies from single-cell RNA-seq
Source: Bioinformatics. 2015 Jun 22;31(20):3380–2. doi: 10.1093/bioinformatics/btv368 (PMC4595899; doi:10.1093/bioinformatics/btv368)
Supplement: Supplementary Data [file supp_31_20_3380__index.html]

Sincell: an R/Bioconductor package for statistical assessment of cell-state hierarchies from single-cell RNA-seq — Supplementary Data 

# *Sincell*: an R/Bioconductor package for statistical assessment of cell-state hierarchies from single-cell RNA-seq

## Supplementary Data

files

- Supplementary Data - pdf file
