# Supplementary material for: Sincell: an R/Bioconductor package for statistical assessment of cell-state hierarchies from single-cell RNA-seq
Source: Bioinformatics. 2015 Jun 22;31(20):3380–2. doi: 10.1093/bioinformatics/btv368 (PMC4595899; doi:10.1093/bioinformatics/btv368)
Supplement: Supplementary Data [file supp_btv368_Sincell_Bioinformatics_Rausell_SuppInfo_v2_online.pdf]

Supplementary Information

Sincell: an R/Bioconductor package for statistical assessment of cell-state hierarchies from single-cell RNA-seq

Miguel Juliá[1,2] , Amalio Telenti[3], Antonio Rausell[1,2*]

[1] Vital-IT group, SIB Swiss Institute of Bioinformatics, 1015 Lausanne, Switzerland

[2] University of Lausanne, 1015 Lausanne, Switzerland

[3] J. Craig Venter Institute, La Jolla, CA 92037

CONTENTS

1 Supplementary Table 1: Computational methods for the assessment of cell-state hierarchies.
2 Supplementary Figure 1: Statistical assessment of cell-state hierarchies in differentiating myeloid cells.
3 Supplementary Text

Method	Reference	Single-cell data	Dimensionality reduction	Metric to assess cell-to-cell distances	Clustering algorithm	Graph -building algorithm / ordering representation	Trajectory assessment	Software availability
**SPADE**	(Qiu *et al.*, 2011)	Mass Cytometry Data and Flow Cytometry Data	NA	L1 distance	Agglomerative clustering	Minimum Spanning Tree (MST)	NA	R/Bioconductor
**Wanderlust**	(Bendall *et al.*, 2014)	Mass Cytometry Data	NA	Cosine distance	NA	K-Nearest Neighbours Graph (K-NNG)	A single non-branching trajectory is assessed from an average of "shortest path"-trajectories over an ensemble of l-out-of-k-nearest-neighbor graphs (l-k- NNGs)	Matlab based
**viSNE**	(Amir *et al.*, 2013)	Mass Cytometry Data and Flow Cytometry Data	t-Distributed Stochastic Neighbor Embedding (t-SNE)	Distance in low-dimensional space	NA	NA	NA	Matlab based
**Monocle**	(Trapnell *et al.*, 2014)	Single-cell RNA-seq	Independent Component Analysis (ICA)	Distance in low-dimensional space	NA	Minimum Spanning Tree (MST)	Longest path through MST is used to define branching trajectories and ordering in "pseudotime"	R/Bioconductor
**Jaitin et al 2014**	(Jaitin *et al.*, 2014)	Single-cell RNA-seq	NA	Correlation	Hierarchical clustering + manual definition of seeds	Circular projection (CAP) of posterior probabilities of association with the model's classes	NA	Not available
**Buettner et al. 2015**	Buettner et al. 2015	Single-cell RNA-seq	Non-linear PCA	Distance in low-dimensional space	Hierarchical clustering with a fix number of clusters on the 2D non-linear PCA plots	NA	NA	Multiple platforms
**SCNS**	Moignard et al 2015	qRT-PCR	Diffusion plot methodology	Distance in low-dimensional space	NA	Discretization of single-cell expression profiles to binary states and connection of those states that differ in the expression of only one gene	NA	F# scripts for Linux, Windows and Mac OS X
**PCA**	(Dalerba *et al.*, 2011; Treutlein *et al.*, 2014)	Single-cell RNA-seq	Principal Component Analysis (PCA)	Distance in low-dimensional space	NA	NA	NA	Multiple platforms

NA: Not Applicable

Supplementary Table 1: Computational methods for the assessment of cell-state hierarchies. The table shows a list of published approaches for the assessment of cell-state heterogeneity from single-cell data together with their main methodological features. The last row includes the standard Principal Component Analysis (PCA) to reflect its use in single-cell data analysis; in this case two references are provided as a non-exhaustive list of examples.

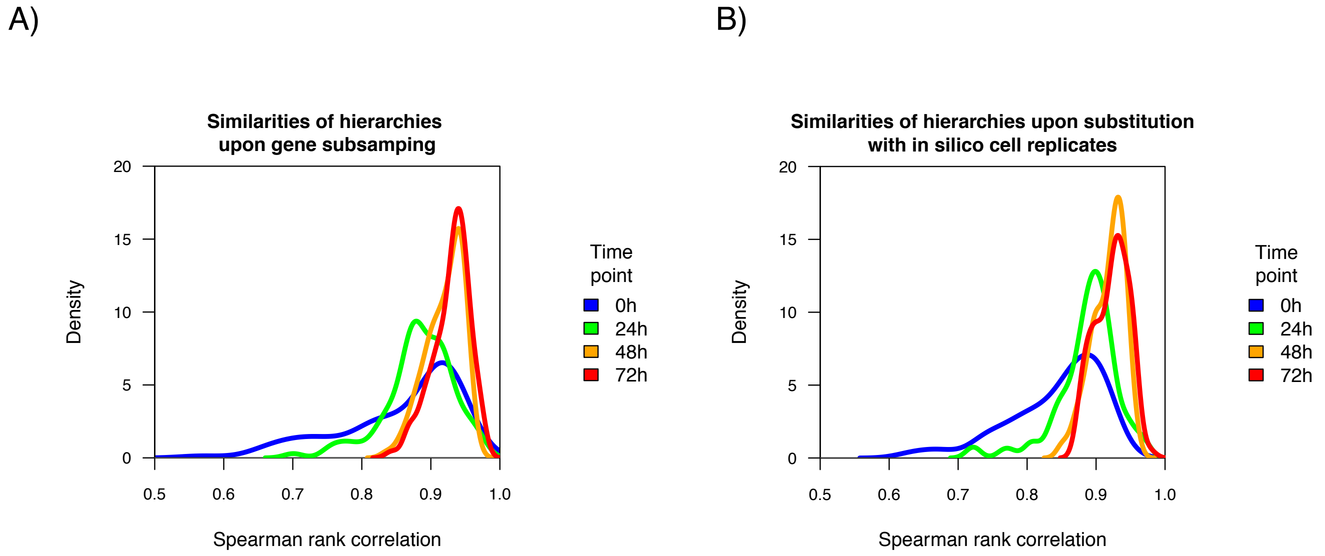

Supplementary Figure 1. Statistical support for cell-state hierarchies obtained in differentiating myoblast samples at 4 timepoints (0, 24, 48 and 72h) from (Trapnell *et al.*, 2014). **A**. Similarities of hierarchies upon random gene subsampling. The figure represents the distribution of similarities between a reference cell-state hierarchy and the 100 hierarchies obtained when 100 random sets of 50% of genes are subsampled. **B**. Similarities of hierarchies upon random cell replacement with *in silico* cell replicates. The figure represents the distribution of similarities between a given cell-state hierarchy and the 100 hierarchies obtained when 100% of individual cells are substituted by a randomly chosen *in silico* replicate. One thousand *in silico* replicates were generated for each cell with default parameters. Four distributions are represented in each panel corresponding to the hierarchies obtained at different timepoints: 0, 24, 48 and 72 hours (blue, green, orange and red respectively). A distribution of similarities with a high median and a low variance is indicative of a cell-state hierarchy robust to variations in the data. See **Supplementary Text** for details.

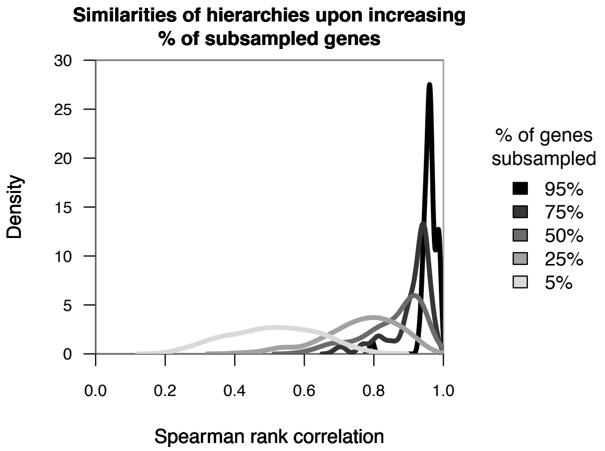

Supplementary Figure 2. Use of Spearman rank correlation between the shortest distance for all pairs of cells is a valid similarity measure between two graphs. The figure shows the distribution of Spearman rank correlation values obtained upon random gene subsampling for increasing numbers of subsampled genes (5%, 25%, 50%, 75% and 95%). Each distribution corresponds to 100 hierarchies obtained when 100 random sets of a given percentage of genes is subsampled. Cell-state hierarchies are assessed for single-cell libraries corresponding to timepoint 0h in Trapnell *et al.*, 2014 on their log-transformed FPKM values using the first two dimensions of a dimensionality reduction with Independent Component Analysis (ICA) and a Minimum Spanning Tree (MST). See **Supplementary Text** for additional details.

SUPPLEMENTARY TEXT

1) Before starting using *Sincell*

The *Sincell* workflow starts from an expression matrix gathering the gene expression levels for every single-cell in the experiment. Before starting to use *Sincell*, quality control to filter out individual cells from the analysis need to be performed by the user. Expression levels also need to be normalized to account for library size or technical variability (e.g. through the use of spike-in molecules). Variance stabilization is also recommended (e.g. through log-transformation).

Some *Sincell* functions are computationally intensive. Implementation of some of *Sincell*'s algorithms in C++ as well as parallelization of *Sincell's* functions decreases run times. However, working with a gene expression matrix of several thousand genes can lead to long computing times depending on the available hardware. If time or computation capacity is an issue, we recommend the user restrict the analysis to the most variable protein coding genes in the dataset. These genes drive most of the signal to assess cell-state hierarchies and restricting the analyses to them should not bias the final results. Typically, running the full set of *Sincell*'s routines on an expression matrix with 2000 genes and 180 individual cells takes less than one hour on a laptop with 8GB RAM. Nevertheless, selecting the most variable genes is not straightforward due to the mean-variance relationship. To select the most variable genes we refer the user to the two following excellent tutorials:

i) the HSC Harvard Catalyst Single-Cell Workshop 2014: RNA-seq (http://pklab.med.harvard.edu/scw2014/subpop_tutorial.html; Section "Identifying highly variable genes")

ii) Bioconductor RNA-Seq workflow (http://master.bioconductor.org/help/workflows/rnaseqGene/#de; Section "The rlog transformation".

Quoting from this last document by Love M, Anders S and Huber W: "*Many common statistical methods for exploratory analysis of multidimensional data, especially methods for clustering and ordination (e.g., principal-component analysis and the like), work best for (at least approximately) homoscedastic data; this means that the variance of an observed quantity (here, the expression strength of a gene) does not depend on the mean. In RNA-Seq data, however, variance grows with the mean. For example, if one performs PCA (principal components analysis) directly on a matrix of normalized read counts, the result typically depends only on the few most strongly expressed genes because they show the largest absolute differences between samples*".

To avoid this bias, we recommend the user perform a variance stabilizing transformation. The simplest way is taking the logarithm of the normalized count values plus 10 (or 100) pseudocounts. More sophisticated strategies are provided by the function *rlog* and other variance stabilizing transformations discussed in the Bioconductor package DESeq2 (http://www.bioconductor.org/packages/release/bioc/vignettes/DESeq2/inst/doc/DESeq2.pdf).

Restricting the analysis to protein coding genes can help further narrow down the number of genes used in the calculations.

Once quality control, normalization and variance stabilization (e.g. log transformation with 100 pseudocounts) have been performed and the most variable genes identified (e.g. the 2000 most variable protein coding genes), we are ready to start working with *Sincell*.

2) Novel graph-building algorithms presented in *Sincell*:

We present here two graph-building algorithms that can be used to infer the progression through a continuum of intermediate cell states: the *Maximum Similarity Spanning Tree* and the *Iterative Mutual Clustering Graph (IMC)*. Both algorithms start from a cell-to-cell distance matrix as an input, and compute a connected graph where nodes represent cells and edges represent their kinship as intermediate cell-states. The weight of an edge connecting two cells corresponds to the original distance between them. The algorithms start with all nodes unconnected, treating them as clusters of size 1.

Maximum Similarity Spanning Tree (SST)

In the first iteration, the two clusters with the lowest distance are connected forming a cluster of size 2. In a new iteration, distances among clusters are recomputed and a new connection is added between the next two clusters with the shortest distance. The distance between a cluster of size higher than one and another cluster is the lowest distance between any of their constituent cells. The process is repeated until there are no cells unconnected.

In contrast to the *Minimum Spanning Tree* (MST) algorithm (that minimizes the total sum of the weights of any possible spanning tree), the SST algorithm prioritizes the highest similarities between any two groups of cells, proceeding in an agglomerative way that represents intermediate cell states. In some cases, MST and SST can lead to the same graph.

Iterative Mutual Clustering Graph (IMC)

In the first iteration, a connection between two clusters A and B is added if A is among the closest *k* nearest clusters of B and B is among the closest *k* nearest clusters of A. This process is iterated until there are no unconnected cells. As with SST, the distance between a cluster of size higher than one and another cluster is the lowest distance between any of their constituent cells.

Note on the assessment of Minimum Spanning Tree algorithms

MST algorithms usually provide a single MST solution. However, in theory there could be multiple, equally optimal solutions. This could happen if several equally parsimonious paths exist or if two or more edges have the same lengths (Salipante and Hall, 2011). Quoting Salipante and Hall 2011: "*In such situations, only one of the possible shortest paths is arbitrarily selected during tree construction, which has the potential to significantly affect the structure of the tree. In light of this issue, it has been appreciated that the solution to the MST problem is better considered a minimum spanning network (MSN), in which all MST solutions are combined into a single graph that demonstrates all equally parsimonious paths*". Nevertheless, in our case the probability of finding several pairs of individual cells with the exact same gene expression profile distance is virtually zero. Therefore, we think this type of potential instability is negligible.

3) Algorithmic strategies to provide statistical support to cell-state hierarchies from single-cell RNAseq

The fact that a cell-state hierarchy is obtained by using any given algorithm (e.g. MST, SST or IMC) does not necessarily imply that it reflects a true biological scenario of cell activation/differentiation. It might well be that the hierarchy obtained is mainly driven by noise due to either biological (Raj *et al.*, 2006; Rand *et al.*, 2012; Shalek *et al.*, 2013; McDavid *et al.*, 2014; Deng *et al.*, 2014) or technical factors (Brennecke *et al.*, 2013; Islam *et al.*, 2014; Grün *et al.*, 2014). The relative contribution of stochastic factors to the observed differences across cells is expected to be higher if cells within a sample are in a homogeneous steady-state. In that case, a low cell-to-cell heterogeneity will lead to cell-state hierarchies very sensitive to small variations in the initial gene expression data. On the other extreme, high levels of cell-to-cell heterogeneity driven by a real granularity in an activation/differentiation process will translate into robust hierarchies that can be reproduced despite stochastic perturbations of the data.

To help discriminate reliable cell-state hierarchies from noisy rearrangements, *Sincell* implements two approaches: i) a strategy relying on a gene resampling procedure and ii) an algorithm based on random cell substitution with *in silico*-generated cell replicates.

A. Gene resampling

This algorithm performs a random subsampling of a given number "*n*" of genes "*s*" times from the original gene expression matrix. For each subsampling, a new connected graph of cells is computed using the same method as for the hierarchy being tested. In each subsampling, the similarity between the resulting graph and the original one is assessed by the Spearman rank correlation between the two graphs of the shortest distance for all pairs of

cells. The distribution of Spearman rank correlation values of all subsamplings can be interpreted as the distribution of similarities between hierarchies that would be obtained from small changes in the data. A distribution with a high median and small variance would indicate a well-supported cell-state hierarchy. On the contrary, a distribution with a low median of similarities and/or a wide variance would indicate a hierarchy very sensitive to changes in the data, and therefore not well statistically supported.

B. Random cell substitution with in silico-generated cell replicates

Gene expression levels detected by single-cell RNA seq are subject to stochastic factors both technical and biological. This means that, if it were possible to profile the same cell in the same cell-state (or, more realistically, a population of individual cells in a highly homogeneous state) multiple times, the detected expression levels of a gene would randomly fluctuate within a distribution of values. In the ideal scenario where that distribution was known for each gene, individual cell replicates could be produced *in silico,* leading to variations in gene expression levels similar to what would be obtained from *in vivo* replicates. The generation of *in silico* replicates would then permit testing the reproducibility of the cell-state hierarchy upon random replacement of a fraction of the original cells with the replicates.

B1. Generation of in silico cell replicates

The distribution of the expression levels of a gene can be described by a measure of variability such as the variance or the coefficient of variation. It is known that the expected variation is dependent on the mean expression values of the gene (Anders and Huber, 2010; Brennecke *et al.*, 2013). Based on this, we can simulate a stochastic fluctuation of the expression of a gene by perturbing the observed level in a given cell with an error term whose magnitude is consistent with the mean-variance relationship observed in the data. By doing that for all genes from an individual cell C_i, we can produce an *in silico* replicate of it.

To this end, *Sincell* implements the following alternative strategies:

i) First, the mean *m* and variance *v* of all genes in the original gene expression matrix is computed. Genes are assigned to classes according to the deciles of the mean to which they belong. Next, for a given gene *g*, a variance *v* is randomly chosen from the set of variances within the class of the gene. Then, a random value drawn from a uniform distribution *U(0,v)* of mean zero and variance *v* is added to the expression value of a gene *g* in a cell *c.* By perturbing all genes in a reference cell *c* in this way, we obtain an *in silico* replicate *c'*. Repeating the process *n* times, *n* stochastic replicates are generated for each original cell.

ii) Alternatively, the strategy described in i) can be modified so that a squared coefficient of variation *cv2* is randomly chosen from the set of coefficient of variation values within the class of the gene. Then, the variance *v* for the uniform distribution is assessed by $v = (cv2 \times m^2)$.

iii) Stochasticity in gene expression at the single-cell level has also been described as following a lognormal distribution *log(x)~N(m,v)* of mean *m* and variance *v* (Bengtsson *et al.*, 2005; Raj *et al.*, 2006). More recently, (Shalek *et al.*, 2014) described gene expression variability in single-cell RNA-seq through a log normal distribution with a third parameter *alpha* describing the proportion of cells where transcript expression was detected above a given threshold. The authors found that the majority of genes in their study (91%) showed distributions well described by the three-parameter model ($p < 0.01$, goodness of fit test; (Shalek *et al.*, 2014)). *Sincell* can use this three parameter model estimation to generate random perturbations of gene expression levels and produce *in silico* cell replicates accordingly.

iv) Other work has found that, for most genes, the variability observed among their expression levels across individual cells was better described by a negative binomial (NB) distribution rather than a lognormal distribution (Grün *et al.*, 2014). Grün and colleagues used NB distribution to model not only technical noise but also true biological gene expression noise. Their assumption was that endoge-

nous mRNA abundance follows a NB as supported by a physical model of bursting expression (Raj *et al.*, 2006). A negative binomial noise model was also adopted in (Zeisel *et al.*, 2015). As pointed out in these works, NB is frequently used to model overdispersed count data and has been previously used for bulk RNA-seq data (Anders and Huber, 2010; Robinson *et al.*, 2010). We recommend this approach only if normalized count data is used (i.e. not length-normalized RPKM/FPKM). *Sincell* can follow an NB distribution parameterized on the observed gene expression levels to generate random perturbations and produce *in silico* cell replicates accordingly. If log-transformed normalized counts are used, *Sincell* would unlog the perturbed data through a NB and afterwards will redo the log transformation.

B2. Random cell substitution with in silico-generated cell replicates

Once cell replicates have been generated, a *Sincell* algorithm performs a random replacement of a given number "*n"* cells in the original gene expression matrix with a randomly selected set of *in-silico* replicates "*s"* times. For each set of substitutions "s", a new connected graph of cells is assessed using the same method as for the hierarchy being tested. In each "s", the similarity between the resulting graph and the original one is assessed as the Spearman rank correlation between the two graphs of the shortest distance for all pairs of cells. The distribution of Spearman rank correlation values of all replacements may be interpreted as the distribution of similarities between hierarchies that would be obtained from stochastic perturbations of a proportion of cells. A distribution with a high median and small variance would indicate a well-supported cell-state hierarchy. On the contrary, a distribution with a low median of similarities and/or a wide variance would indicate a hierarchy very sensitive to changes in the data, and therefore not well statistically supported.

C. Use of spike-in molecules to deconvolute technical and biological noise.

The use of spike-in molecules is recommended in single-cell RNA-seq to infer the amount of variability in the expression levels of one gene that is expected to arise from technical factors (Brennecke *et al.*, 2013; Grün *et al.*, 2014; Islam *et al.*, 2014). More recently, Ding et al. went a step further by using spike-ins to explicitly remove technical noise and compute de-noised gene expression levels (R software GRM, http://wanglab.ucsd.edu/star/GRM/ , (Ding *et al.*, 2015). When spike-ins are available, we recommend first performing a technical de-noise procedure on the expression matrix before using *Sincell*. In such a way, Sincell's computations of cell-state hierarchies would mainly rely on biological variation.

Notwithstanding, biological variation will contain two main components. First, the heterogeneity explained by truly different cell-states in a differentiation/activation process, which is the component we aim to capture in a hierarchy. Second, the intrinsic biological noise that is expected to arise even among cells in the same differentiation/activation state. Intrinsic biological noise originates from the characteristics of gene expression mechanisms such as bursts of transcription (Raj *et al.*, 2006), the stochasticity of signal transmission (Rand *et al.*, 2012), bimodality (Shalek *et al.*, 2013), cell cycle effects (McDavid *et al.*, 2014; Buettner *et al.*, 2015) and random monoallelic expression (Deng *et al.*, 2014).

Therefore, even if technical noise has been removed, it is important to test whether the hierarchy is mainly determined by real cell-state heterogeneity or, on the contrary, it is an artifact of the intrinsic biological noise. To this end, the reproducibility of the cell-state hierarchy can be evaluated upon random perturbations of de-noised expression levels. *Sincell*'s strategies described above based on either i) random gene subsampling or ii) random cell substitution with *in silico*-generated cell replicates, would in that case test whether the hierarchy is robust to stochastic perturbations of the data mimicking the effect of intrinsic biological noise.

Additionally, the user may eventually wish to test the reproducibility of the hierarchy assessed on the original expression data (i.e. not first performing a technical de-noising step) upon random perturbations based exclusively on technical noise. Estimates of technical noise for each gene can be obtained by modeling the dependence of the coefficient of variation (cv2) of spike-in molecules as a function of their average expression. For instance, in Brennecke et al. 2013, for each technical gene i (e.g. the spike-ins), the sample mean (m) and sample variance of its normalized counts are estimated. Then, the observed squared coefficients of variation (cv2) are fitted against

the sample mean (m) with a generalized linear model of the gamma family with identity link and parameterization cv2=$\tilde{a}_1$/m+ α_0. Applying the fitted formula to the sample mean expression levels of a gene provides an estimate of cv2 arising from technical noise. *Sincell* permits the incorporation of a technical cv2 estimate per gene in the assessment of *in silico* cell replicates based on normalized counts (i.e. following the previously described negative binomial distribution whose dispersion is parameterized using the estimated technical cv2). Alternatively, in the absence of spike-in molecules, *Sincell* implements the fit described in Brennecke et al. 2013 using the cv2 and m values of all genes in the input expression matrix to provide a surrogate of technical noise estimates. However, this alternative should not be used if the user has previously followed our recommendation in **Section 1** of using such an approach to identify highly variable genes in order to decrease the size of the input matrix (http://pklab.med.harvard.edu/scw2014/subpop_tutorial.html; Section "Identifying highly variable genes").

D. Application of Sincell *algorithms to provide cell-state hierarchies with statistical support on a real single-cell RNA seq data set*

We applied *Sincell* algorithms to provide cell-state hierarchies with statistical support to a publicly available single-cell RNA-seq dataset from (Trapnell *et al.*, 2014). The authors generated single-cell RNA-seq libraries for differentiating myoblasts at 0, 24, 48 and 72 hours. Original data can be accessed at GEO database accession number GSE52529 (ftp://ftp.ncbi.nlm.nih.gov/geo/series/GSE52nnn/GSE52529/suppl/GSE52529_fpkm_matrix.txt.gz). Following (Trapnell *et al.*, 2014) and the vignette of its associated Bioconductor package *Monocle* (http://www.bioconductor.org/packages/devel/bioc/html/monocle.html), the expression matrix is restricted to 575 genes differentially expressed between cells from time 0 and the ensemble of cells of times 24, 48 and 72 hours of differentiation. Here, we analyze each time-point separately and evaluate the statistical evidence of cell-state heterogeneity within them.

Four cell-state hierarchies were assessed for each timepoint separately (0, 24, 48 and 72h) on their log-transformed FPKM values using the first two dimensions of a dimensionality reduction with Independent Component Analysis (ICA) and a Minimum Spanning Tree (MST). To evaluate the statistical support of the arrangements obtained, two *Sincell* algorithms were applied: i) a gene resampling procedure and ii) a random cell substitution with *in silico*-generated cell replicates. **Supplementary Figure 1A** represents the distribution of similarities between a reference cell-state hierarchy and the 100 hierarchies obtained when a random set of 50% of genes are subsampled 100 times. **Supplementary Figure 1B** represents the distribution of similarities between a reference cell-state hierarchy and the 100 hierarchies obtained when 100% of the cells are replaced by a randomly chosen *in silico* replicate 100 times. Here we used a uniform distribution as described in Section 3.B.2.i)

In both cases, late timepoints lead to hierarchies with a high median while early time points had a lower median and a higher variance. Results suggest that at early time points homogeneity of cell states is high, leading to hierarchies more sensitive to perturbations of the data and therefore less statistically supported. However, late timepoints showed hierarchies more robust to both gene subsampling and replacement with *in-silico* replicates, reflecting a marked heterogeneity in cell states. Indeed, a gradient can be observed in both panels (from 0 to 24, 48 and 72h) suggesting that heterogeneity in cell-states increased as a function of time.

E. Note on the use of the Spearman rank correlation between the shortest distance for all pairs of cells as a similarity measure between two graphs. This way of comparing graphs is taken from a similar approach used to compare phylogenetic trees (Pazos *et al.*, 2005; Juan *et al.*, 2008) or against positions within a Multiple Sequence Alignment (del Sol Mesa *et al.*, 2003). To provide evidence of its use as a valid similarity measure between graphs we assessed the distribution of Spearman rank correlation values obtained between a reference graph and a population of 100 hierarchies obtained when 100 random sets of a given percentage of genes are subsampled 100 times. We did this for increasing percentages of subsampled genes: 5%, 25%, 50%, 75% and 95%. **Supplementary Figure 2** shows these distributions reflecting the expected behavior, that is: the more representative the subsampling (higher number of sampled genes) the higher the similarity of the resulting hierarchies with the reference.

Other metrics to compare graphs that could have been used include e.g.: i) the symmetric difference of two sets of edges, i.e. the set of edges present in either of the sets but not in their intersection. This distance evaluates the

topology of the graphs, but does not consider distances between nodes. And ii) least moves, i.e. the minimum number of changes that graph A needs to become graph B. This distance is useful to measure difference between topologies, and weighted edges can be used giving different values to the actions of resizing, removing or adding an edge (See Boorman and Olivier, 1973 for a discussion on this metrics). However, we decided to use our approach based on rank correlations because it is less sensitive to small variations in the topology as far as they occur in the vicinity (i.e. they don't translate in large path distances between the affected nodes).

4) Functional association tests to help interpret cell-state hierarchies

Once a cell-state hierarchy has been assessed and its statistical support checked, the next step is interpreting the hierarchy in functional terms. *Sincell* allows different graphical representations that can help interpret the hierarchies in terms of the features of the samples (e.g. differentiation time) or the expression levels of markers of interest. In this section, we propose an analytical approach to test whether the cell-state hierarchy associates with a given functional gene set, that is: whether the relative similarities among the individual cells in the hierarchy are driven by the expression levels of a subset of genes with a common functional feature.

Sincell implements an algorithm to evaluate this association. First, a new cell-state hierarchy is assessed where only the expression levels of the genes in a given functional gene set are considered. Second, the similarity of that hierarchy with the reference hierarchy (the one assessed on the initial gene expression matrix) is calculated. The similarity between the two hierarchies is computed as the Spearman rank correlation between the two graphs of the shortest distance for all pairs of cells. Third, an empirical p-value of the observed similarity between the two hierarchies is provided. The empirical p-value is derived from a distribution of similarities resulting from random samplings of gene sets of the same size.

This *Sincell* algorithm is particularly suited to evaluate associations with gene set collections such as those from the Molecular Signatures Database (MSigDB) of the Broad Institute (http://www.broadinstitute.org/gsea/msigdb/collections.jsp), gene lists representing Gene Ontology terms of functional pathways, and in general, any gene set collections that might be of particular interest for the user.

REFERENCES

Amir,E.D. *et al.* (2013) viSNE enables visualization of high dimensional single-cell data and reveals phenotypic heterogeneity of leukemia. *Nat. Biotechnol.*, **31**, 545–552.

Anders,S. and Huber,W. (2010) Differential expression analysis for sequence count data. *Genome Biol.*, **11**, R106.

Bendall,S.C. *et al.* (2014) Single-Cell Trajectory Detection Uncovers Progression and Regulatory Coordination in Human B Cell Development. *Cell*, **157**, 714–725.

Bengtsson,M. *et al.* (2005) Gene expression profiling in single cells from the pancreatic islets of Langerhans reveals lognormal distribution of mRNA levels. *Genome Res.*, **15**, 1388–1392.

Boorman,S.A. and Olivier,D.C. (1973) Metrics on spaces of finite trees. *J. Math. Psychol.*, **10**, 26–59.

Brennecke,P. *et al.* (2013) Accounting for technical noise in single-cell RNA-seq experiments. *Nat. Methods*, **10**, 1093–1095.

Buettner,F. *et al.* (2015) Computational analysis of cell-to-cell heterogeneity in single-cell RNA-sequencing data reveals hidden subpopulations of cells. *Nat. Biotechnol.*, **33**, 155–160.

Dalerba,P. *et al.* (2011) Single-cell dissection of transcriptional heterogeneity in human colon tumors. *Nat Biotech*, **29**, 1120–1127.

Deng,Q. *et al.* (2014) Single-Cell RNA-Seq Reveals Dynamic, Random Monoallelic Gene Expression in Mammalian Cells. *Science*, **343**, 193–196.

Ding,B. *et al.* (2015) Normalization and noise reduction for single cell RNA-seq experiments. *Bioinformatics*, btv122.

Grün,D. *et al.* (2014) Validation of noise models for single-cell transcriptomics. *Nat. Methods*, **11**, 637–640.

Islam,S. *et al.* (2014) Quantitative single-cell RNA-seq with unique molecular identifiers. *Nat. Methods*, **11**, 163–166.

Jaitin,D.A. *et al.* (2014) Massively Parallel Single-Cell RNA-Seq for Marker-Free Decomposition of Tissues into Cell Types. *Science*, **343**, 776–779.

Juan,D. *et al.* (2008) High-confidence prediction of global interactomes based on genome-wide coevolutionary networks. *Proc. Natl. Acad. Sci.*, **105**, 934–939.

McDavid,A. *et al.* (2014) Modeling Bi-modality Improves Characterization of Cell Cycle on Gene Expression in Single Cells. *PLoS Comput Biol*, **10**, e1003696.

Pazos,F. *et al.* (2005) Assessing Protein Co-evolution in the Context of the Tree of Life Assists in the Prediction of the Interactome. *J. Mol. Biol.*, **352**, 1002–1015.

Qiu,P. *et al.* (2011) Extracting a cellular hierarchy from high-dimensional cytometry data with SPADE. *Nat. Biotechnol.*, **29**, 886–891.

Raj,A. *et al.* (2006) Stochastic mRNA Synthesis in Mammalian Cells. *PLoS Biol*, **4**, e309.

Rand,U. *et al.* (2012) Multi-layered stochasticity and paracrine signal propagation shape the type-I interferon response. *Mol. Syst. Biol.*, **8**.

Robinson,M.D. *et al.* (2010) edgeR: a Bioconductor package for differential expression analysis of digital gene expression data. *Bioinformatics*, **26**, 139–140.

Salipante,S.J. and Hall,B.G. (2011) Inadequacies of Minimum Spanning Trees in Molecular Epidemiology. *J. Clin. Microbiol.*, **49**, 3568–3575.

Shalek,A.K. *et al.* (2014) Single-cell RNA-seq reveals dynamic paracrine control of cellular variation. *Nature*, **510**, 363–369.

Shalek,A.K. *et al.* (2013) Single-cell transcriptomics reveals bimodality in expression and splicing in immune cells. *Nature*.

Del Sol Mesa,A. *et al.* (2003) Automatic Methods for Predicting Functionally Important Residues. *J. Mol. Biol.*, **326**, 1289–1302.

Trapnell,C. *et al.* (2014) The dynamics and regulators of cell fate decisions are revealed by pseudotemporal ordering of single cells. *Nat. Biotechnol.*, **32**, 381–386.

Treutlein,B. *et al.* (2014) Reconstructing lineage hierarchies of the distal lung epithelium using single-cell RNA-seq. *Nature*, **509**, 371–375.

Zeisel,A. *et al.* (2015) Cell types in the mouse cortex and hippocampus revealed by single-cell RNA-seq. *Science*, **347**, 1138–1142.
